# Editorial: Healthcare organization and delivery strategies, models, and cost savings

**DOI:** 10.3389/fpubh.2023.1255493

**Published:** 2023-08-21

**Authors:** Haichang Xin

**Affiliations:** Independent Researcher, Birmingham, AL, United States

**Keywords:** holistic approach, healthcare organization and delivery, strategies, models, healthcare costs

Healthcare costs continue to grow, which has imposed a heavy burden on global healthcare systems. Innovative healthcare organization and delivery strategies and models are one mechanism to deal with rising costs since they aim to improve healthcare quality and access, reduce the unnecessary duplication of services, and prevent medical errors.

As such, the journal *Frontiers in Public Health, Health Economics*, has organized a special topic on *Healthcare Organization and Delivery Strategies, Models, and Cost Savings* as it is directly related to healthcare access, quality, and costs. This edition has called for articles to explore reasons at different levels and suggest promising interventions to reduce costs and improve healthcare access and quality. All the submissions have gone through the peer review process. By the time of submission closing, we accepted five articles for publication.

These studies cover various healthcare challenges, such as high-cost persistence, hospitalization expenses, poverty-driven healthcare use and costs, social determinants of health utilization, and limited access to healthcare.

These studies have used different data, such as electronic medical records, public health insurance program data, and systematic reviews of literature to identify trends, patterns, and factors related to the respective healthcare challenges.

These studies collectively highlight the multifaceted nature of healthcare challenges in healthcare costs, use, and access, and the diverse approaches in healthcare organization, delivery, and finance being taken to address those challenges. They have identified influencing factors such as health status, family support, age, length of hospital stay, comorbidities, treatment mode, and technology for these challenges. Overall, these findings provide insights and recommendations for policymakers, healthcare providers, and researchers to design effective strategies and interventions to tackle healthcare challenges in different contexts. These interventions would be based on the identified influencing factors.

For the healthcare delivery domain, some studies suggest the need for targeted interventions and innovative delivery models to improve access to care, such as leveraging advanced technologies like telehealth for enhanced communication and healthcare access. Other studies suggest improving health status and family supports to reduce costs or call for greater use of social determinants of health in electronic medical records. For the healthcare finance domain, some studies suggest the potential of integrated microfinance and health initiatives in providing financial support, access to affordable medicine, and protection against out-of-pocket health expenses. Another study suggests developing personalized and precise compensation mechanisms based on factors such as treatment mode for specific conditions to control healthcare expenditures.

Multifaceted challenges are usually related to one another. For example, many persistent high costs may come from inpatient costs, and limited access to healthcare may be associated with limited access to social resources in the domain of social determinants of health.

Facing such complex and multifaceted healthcare challenges in healthcare utilization and costs, policymakers can consider a holistic approach to deal with them, using innovative healthcare organization, delivery, and finance strategies and models. Rather than focusing on isolated issues or individual challenges, a comprehensive perspective and holistic view of multifaceted challenges will focus on combined or integrated interventions, which may address multiple challenges simultaneously. Thus, a holistic approach is more efficient. Moreover, a holistic approach recognizes the interconnectedness of various components in healthcare systems, emphasizing the importance of interdisciplinary collaboration, efficient allocation of resources, and alignment of interaction between different stakeholders, such as healthcare providers, policymakers, and patients.

In conclusion, these studies have discussed specific topics from different perspectives in healthcare costs, utilization, and access. As a whole, they collectively inform policies of meaningful interventions using innovative healthcare organization, delivery, and finance strategies and models. Starting from there, a holistic approach can better synthesize these suggested interventions to enhance the efficiency and effectiveness of healthcare organization, delivery, and finance, ultimately leading to increased healthcare quality and access and cost savings.

## Author contributions

HX: Conceptualization, Writing–original draft, Writing–review and editing.

